# A case of subarachnoid hemorrhage revealed by an acute coronary syndrome (ACS)

**DOI:** 10.11604/pamj.2015.20.426.4741

**Published:** 2015-04-29

**Authors:** Abdedaim Hatim, Wafae El Otmani, Mehdi Ait Houssa, Noureddine Atmani, Younes Moutakiallah, Charqui Haimeur, Mohammed Drissi

**Affiliations:** 1 Cardiovascular Surgery Department, Military Hospital Mohammed V, Rabat, Morocco; 2 Medical Intensive Care Department, Military Hospital Mohammed V, Rabat, Morocco

**Keywords:** Subarachnoid hemorrhage, acute coronary syndrome, acute neurogenic pulmonary edema, cardiogenic shock, catecholamine discharge

## Abstract

The subarachnoid hemorrhage (SAH) is definitely the best descriptive model of the interaction between cardiovascular system and cerebral damage. The underlying mechanism of cardiovascular alterations after SAH is linked to the adrenergic discharge related to aneurysm rupture. Cardiac and pulmonary complications are common after severe brain injury, especially the aneurismal subarachnoid hemorrhage. Acute neurogenic pulmonary edema is not exceptional; it may occur in 20% of cases and commonly follows a severe subarachnoid hemorrhage. Severe myocardial damage with cardiogenic shock may possibly reveal the SAH (3% of cases) and mislead to wrong diagnosis of ACS with dramatic therapeutic consequences. The contribution of CT and cerebral angiography is essential for diagnosis and treatment. Surgical or endovascular treatment depends on location, size and shape of the aneurysm, on patient's age, neurological status and existence of concomitant diseases. We report the case of a 58 years old patient, with a past medical history of diabetes and hypertension, admitted for acute pulmonary edema with cardiogenic shock. This case illustrates an unusual presentation of aneurismal SAH in a patient presenting with an acute coronary syndrome.

## Introduction

Cardiac and pulmonary complications are common after severe brain injury such as aneurismal SAH and are associated with increased mortality and morbidity. Acute neurogenic pulmonary edema has been reported in up to 20% of patients after severe SAH, 3% of which have a severe myocardial damage and a cardiogenic shock. The most accepted theory for cardiac dysfunction is the “catecholamine hypothesis” in which brain damage in aneurismal SAH leads to adrenergic discharge. We report the case of a 58-year-old patient with a past medical history of diabetes and hypertension, admitted to the ICU for acute pulmonary edema with cardiogenic shock after acute myocardial infarction with significant troponin rise and ST-segment elevation. We'll emphasize the challenge in diagnosing SAH in such clinical situations and point out cardiovascular manifestations of subarachnoid hemorrhage and the principles of management of these patients.

## Patient and observation

**Figure 1:**
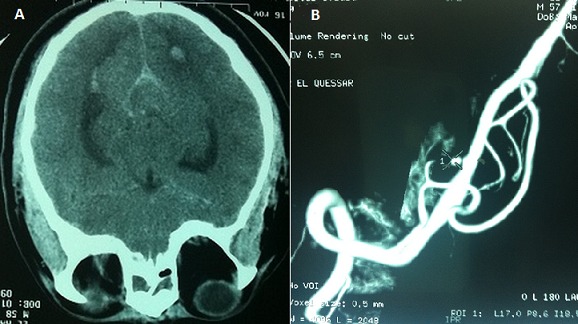
(A) Brain CT: ventricular flood with spontaneous hyperdensity of sulci and sylvian valleys; (B): magnetic resonance angiography (MRA): aneurysm of the initial segment of posterior inferior cerebellar artery (PICA)

**Figure 2:**
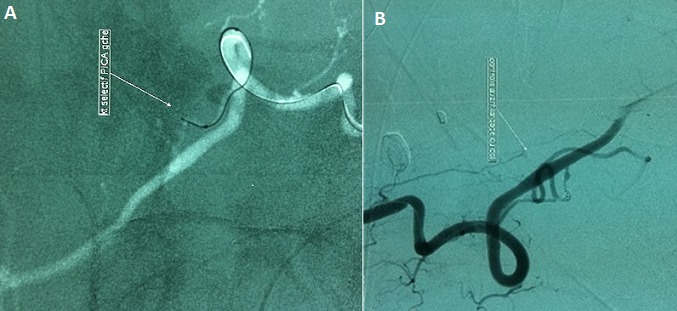
(A) MRA: angiographic embolization with microspheres of the aneurysm; (B): postembolization angiography

A 58-year-old man with a past medical history of poorly controlled hypertension and type 2 diabetes, presented to the ICU for a cardiogenic shock complicating acute myocardial infarction. Physical examination revealed reduced level of consciousness (Glasgow Coma Scale 10/15) and weak vital signs; a blood pressure of 80/50 mmHg, 80% of oxygen saturation, capillary blood glucose at 2.26 g/L, a heart rate over 125 bpm, and crackling in pulmonary auscultation. Cardiovascular examination showed neither cardiac murmur nor signs of right heart failure. ECG on admission showed normal sinus rhythm, with heart rate of 125 bpm and extended ST-elevation in anterior territory. Laboratory results demonstrated Troponin I level of 6.41 ng/ml, creatinine kinase (CKMB) was 67 UI/L, Lactate deshydrogenase was 281 UI/L, glucose level 2.70 g/l, urea 0.40 g/l and creatinine 18.6 mg / L. The patient was intubated and sedated, inotropic agents were started (norepinephrine 0.4µg/kg/min and dobutamine 20µg/kg/min). Chest X-rays showed diffuse alveolar syndrome. Transthoracic echocardiography revealed wall motion abnormalities namely extensive akinesis of anteroseptal, anterior, lateral and inferior walls, and severe left ventricular systolic dysfunction (ejection fraction of 29%). Medical management was initiated; anticoagulant therapy for acute coronary syndrome was started (500 mg of acetylsalicylic acid and subcutaneous low-molecular-weight heparin (0.6ml of enoxaparin)) and patient was prepared for myocardial revascularization by coronary angioplasty. Because of non-improvement of neurological status and occurrence of seizures, a brain CT was indicated and revealed infratentorial diffuse hemorrhage (Fisher grade III) (Figure 1). Cerebral angiography confirmed a dissecting aneurysm of an anastomotic branch between left PICA and the V4 segment of left vertebral artery Figure 2 that was successfully embolized. After 24 hours, the patient improved with withdrawal of vasoactive drugs in 24 hours. Left hemicorporeal seizures persisted despite anticonvulsant treatment. Control brain CT did not report rebleeding and angiography showed complete exclusion of the aneurysm while the EEG revealed a diffuse brain damage. 10 days after admission, the patient was discharged in stable condition but still suffers from amnesia.

## Discussion

Cardiovascular control centers are mainly located in the brain stem and hypothalamus. The tract of the solitary nucleus is the most important integration center for circulatory control. Autopsy studies show that cardiac damage during acute neurological pain is important with a high frequency of transmural myocardial damage [1]. Cardiovascular and pulmonary complications occur frequently after aneurismal SAH. Acute neurogenic pulmonary edema is not exceptional; it may occur early in 20% of cases and frequently accompanies severe subarachnoid hemorrhage [2]. Severe myocardial lesions with cardiogenic shock can also reveal SAH misleading the diagnosis to acute coronary syndrome with dramatic therapeutic consequences as was the case of our patient. Different pathophysiological mechanisms in neurogenic pulmonary oedema have been suggested. These mechanisms are increase in capillary hydrostatic pressure because of catecholamine discharge and post capillary vasoconstriction, increase of alveolar-capillary membrane permeability and acute heart failure [3]. Clinical presentation is made of pulmonary edema with hypoxemia and hematosis disorders that may exacerbate brain damage. Clinical manifestations of cardiac dysfunction in SAH are similar to others situations caused by massive catecholamine release, such as tako-tsubo cardiomyopathy that is characterized by reversible wall motion abnormalities, elevation in biological myocardial markers and transitory ST-T changes, in the absence of coronary artery disease [4, 5]. ECG abnormalities in patients with SAH are variable, including ST-segment elevation or depression, pathologic Q waves and arrhythmias [6–10].

The cause of ST-segment elevation accompanying SAH is the stimulation of the anterior hypothalamus. Ischemic stimulation of the posterior hypothalamus seems to be responsible for an increase in sympathetic tone and catecholamine discharge [6]. Coronary artery stenosis is not necessarily found in patients with SAH [11, 12]. Hypotension is rarely seen in SAH [13]. It's caused by peripheral arterial vasodilatation; hypovolemia is associated in 50% of cases. It may also result from cardiac dysfunction. Tako-tsubo cardiomyopathy or apical ballooning syndrome is a rare cause of ventricular dysfunction after SAH but increases mortality in this context [14, 15]. Hypotension and hypoxia are the main secondary brain insults of systemic origin. Enzymatic increase with high values of troponin I (20% to 68% according to different surveys) is correlated to a poor prognosis. ECG alterations such as ST segment, T wave or Q wave abnormalities are very common but nonspecific (40 to 70% of patients). Life-threatening arrhythmias, including ventricular ones, occur in a 5 to 8% of patients [14, 16]. The combination of acute respiratory distress, shock, electrical modifications, biomarkers’ elevation and global myocardial dysfunction misled the diagnosis to acute coronary syndrome with acute pulmonary edema and cardiogenic shock for which medical management requires effective anticoagulation based on antiplatelet agents (acetylsalicylic acid) and low molecular weight heparin (LMWH). This treatment is seriously dangerous because it increases risk of bleeding (in this context an unknown subarachnoid hemorrhage).

In fact, in the present case, neurological disorders indicated a brain CT and cerebral angiography that confirmed the SAH diagnosis. Although complications are associated with adverse outcome, there is still a paucity of evidence on their optimal management. Treatment of cardiac complications in SAH is mainly supportive as the condition generally improves over time. When Congestive heart failure and pulmonary congestion occur, goal-directed haemodynamic monitoring is justified. Optimization of cerebral perfusion and oxygen delivery in SAH requires haemodynamic support in patients with reduced cardiac output. Treatment with inotropic agents such as dobutamine is beneficial to boost cardiac performance [17]. Norepinephrine can also be used to maintain cerebral perfusion pressure (CPP), but this treatment presents two serious complications in this context: the increase in CPP before diagnostic and therapeutic angiography exposes to major risk of rebreeding; Alterations in myocardial function concomitant to cerebral artery vasospasm that requires triple H therapy (hypertension, hemodilution and hypervolemia). The choice between endovascular and microsurgical therapy depends on location, size, and shape of the aneurysm, patient's age, comorbidities and neurological status. The ongoing evaluation of aneurysm occlusion techniques is necessary to choose the optimal treatment for each case. New materials and endovascular technologies, as well as combined intraoperative angiography or combined interventions are very promising future treatment of cerebral aneurysms.

## Conclusion

Aneurismal subarachnoid hemorrhage may present with serious cardiac and pulmonary disturbances. Cardiac dysfunction and pulmonary edema can reveal a SAH misleading the diagnosis to acute coronary syndrome with dramatical therapeutic consequences. Cardiac abnormalities in SAH are often transient and their management should focus on general supportive care and treatment of injured brain. Further studies are necessary to explore treatment options and to establish whether there is a causal relationship between cardiac dysfunction and neurological prognosis.

## References

[1] Doshi R, Neil-Dwyer G (1977). Hypothalamic and myocardial lesions after subarachnoid haemorrhage. J Neurol Neurosurg Psychiatry..

[2] Mertes P-M, Bruder N, Audibert G (2012). Complications cardiovasculaires de l'agression cerebrale. Annales Françaises d'Anesthésie et de Réanimation..

[3] Masuda T, Sato K, Yamamoto S, Matsuyamal N, Shimohama T, Matsunaga A (2002). Sympathetic nervous activity and myocardial damage immediately after subarachnoid hemorrhage in a unique animal model. Stroke..

[4] Elrifia AM, Bailes JE, Shih SR (1996). Characterization of cardiac effects of acute subarachnoid hemorrhage in dogs. Stroke..

[5] Zaroff JG, Rordorf GA, Titus JS, Newell JB, Nowak NJ, Torchiana DF, Aretz HT, Picard MH, Macdonald RL (2000). Regional myocardial perfusion after experimental subarachnoid hemorrhage. Stroke..

[6] Davis TP, Alexander J, Lesch M (1993). Electrocardiographic changes associated with acute cerebrovascular disease: a clinical review. Prog Cardiovasc Dis..

[7] Zaroff JG, Rordorf GA, Newell JB, Ogilvy CS, Levinson JR (1999). Cardiac outcome in patients with subarachnoid hemorrhage and electrocardiographic abnormalities. Neurosurgery..

[8] Brouwers PJ, Wijdicks EF, Hasan D (1989). Serial electrocardiographic recording in aneurysmal subarachnoid hemorrhage. Stroke..

[9] Melin J, Fogelholm R (1983). Electrocardiographic findings in subarachnoid hemorrhage: a population study. Acta Med Scand..

[10] Carruth JE, Silverman ME (1980). Torsade de pointe atypical ventricular tachycardia complicating subarachnoid hemorrhage. Chest..

[11] Sakamoto H, Nishimura H, Imataka K, Ieki K, Horie T, Fujii J (1996). Abnormal Q wave, ST-segment elevation, T-wave inversion, and widespread focal myocytolysis associated with subarachnoid hemorrhage. Jpn Circ J..

[12] Nakamura Yl, Kaseno K, Kubo T (1989). Transient ST-segment elevation in subarachnoid hemorrhage. J Electrocardiol..

[13] Kassell NF, Torner JC, Haley EC (1990). The International Cooperative Study on the Timing of Aneurysm Surgery, part 1: Overall management results. J Neurosurg..

[14] Behrouz R, Sullebarger JT, Malek AR (2011). Cardiac manifestations of subarachnoid hemorrhage. Expert Rev Cardiovasc Ther..

[15] Temes RE, Tessitore E, Schmidt JM, Naidech AM, Fernandez A, Ostapkovich ND (2010). Left ventricular dysfunction and cerebral infarction from vasospasm after subarachnoid hemorrhage. Neurocrit Care..

[16] Papanikolaou J, Makris D, Karakitsos D, Saranteas T, Karabinis A, Kostopanagiotou G (2012). Cardiac and central vascular functional alterations in the acute phase of aneurysmal subarachnoid hemorrhage. Crit Care Med..

[17] Naidech A, Du Y, Kreiter KT, Parra A, Fitzsimmons BF, Lavine SD (2005). Dobutamine versus milrinone after subarachnoid hemorrhage. Neurosurgery..

